# A global, cross-sectional survey of patient-reported outcomes, disease burden, and quality of life in epidermolysis bullosa simplex

**DOI:** 10.1186/s13023-022-02433-3

**Published:** 2022-07-15

**Authors:** Jodi Y. So, Shivali Fulchand, Christine Y. Wong, Shufeng Li, Jaron Nazaroff, Emily S. Gorell, Mark P. de Souza, Dedee F. Murrell, Joyce M. Teng, Albert S. Chiou, Jean Y. Tang

**Affiliations:** 1 Department of Dermatology, Stanford University School of Medicine, Stanford, CA USA; 2 Department of Urology, Stanford University School of Medicine, Stanford, CA USA; 3 Department of Dermatology, University of Cincinnati, College of Medicine, Cincinnati, OH USA; 4 Phoenix Tissue Repair, Inc., Boston, MA USA; 5 Department of Dermatology, University of New South Wales, Sydney, NSW Australia

**Keywords:** Epidermolysis bullosa simplex, Epidermolysis bullosa, Genodermatoses, Bullous dermatoses, Quality of life, Patient-reported outcomes

## Abstract

**Background:**

Epidermolysis bullosa simplex (EBS) comprises a group of rare, blistering genodermatoses. Prior work has been limited by small sample sizes, and much remains unexplored about the disease burden and health-related quality of life (QOL) of patients with EBS. The aim of this study was to characterize the most common patient-reported clinical manifestations and the health-related impact of QOL in EBS, and to examine differences in disease burden by age.

**Methods:**

Patients with a diagnosis of epidermolysis bullosa (EB) or their caregivers completed a one-time online survey administered by EBCare, an international online EB registry. Survey data from respondents self-reporting a diagnosis of EBS were analyzed for clinical and wound manifestations, medication use, and QOL (using Quality of Life in Epidermolysis Bullosa [QOLEB] scores). Differences across age groups were assessed using Kruskal–Wallis and Fisher’s exact tests.

**Results:**

There were 214 survey respondents with EBS. The mean age was 32.8 years (standard deviation = 19.2). Many respondents reported blisters (93%), recurrent wounds (89%), pain (74%), chronic wounds (59%), itch (55%), and difficulty walking (44%). Mean QOLEB score was 14.7 (standard deviation = 7.5) indicating a “moderate” impact on QOL, and 12% of respondents required regular use of opiates. Findings were consistent in subgroup analyses restricted to respondents with diagnostic confirmation via genetic testing or skin biopsy (n = 63 of 214). Age-stratified analyses revealed differences in disease burden: younger respondents were more likely to self-report severe disease (24% vs. 19% vs. 5% for respondents aged 0–9 vs. 10–17 vs. 18 + , p = 0.001), failure to thrive (9% vs. 15% vs. 3%, p = 0.02), and use of gastrostomy tubes (15% vs. 12% vs. 1%, p < 0.001) and topical antibiotics (67% vs. 69% vs. 34%, p < 0.001), while older respondents were more likely to be overweight or obese (6% vs. 0% vs. 51%, p < 0.001) and have difficulty walking (24% vs. 46% vs. 48%, p = 0.04).

**Conclusions:**

In the largest international cross-sectional survey of EBS patients conducted, respondents reported extensive disease burden including significant wounding, pain, itch, difficulty walking, and impact on QOL. Age stratified disease manifestations. These findings suggest significant unmet need, and treatment and counseling for EBS patients should consider age-specific differences.

**Supplementary Information:**

The online version contains supplementary material available at 10.1186/s13023-022-02433-3.

## Background

Epidermolysis bullosa simplex (EBS) is a diverse group of rare genodermatoses characterized by intraepidermal blistering and skin fragility due to intracytoplasmic cleavage of basal keratinocytes [1]. EBS is typically inherited in an autosomal dominant manner, or may be a spontaneous mutation, and is most often caused by mutations in keratin 5 (*KRT5*) and keratin 14 (*KRT14*) which form intracellular keratin filaments and are critical for maintaining cell structure and integrity following mechanical trauma [2–5]. EBS is the most common type of epidermolysis bullosa (EB), accounting for approximately 70% of all cases [4]. In the US, the prevalence of EBS was previously estimated at 6 per million with an incidence of 8 per million live births [6]. Estimates may vary across different populations, however, with one recent study of Dutch patients identifying a prevalence of 12 per million and incidence of 17.5 per million live births [7]. The most recent consensus reclassification identifies 14 clinical subtypes associated with seven distinct genes, with phenotypes ranging widely from mild blistering on the hands and feet (i.e., localized EBS, formerly EBS Weber-Cockayne), to large generalized blisters (i.e., severe EBS, formerly EBS-Dowling-Meara or EBS-generalized severe), to extracutaneous involvement and premature death (i.e., severe EBS with pyloric atresia) [1, 8, 9]. Previous research also suggests that the clinical manifestations of EBS may evolve over time, with the potential for decreased severity and frequency of blistering but increased risk of obesity and difficulty walking as patients reach adolescence and adulthood [10, 11].

This phenotypic diversity and potential for severe disease calls into question the widely held notion of EBS as simply a mild variant of EB. Prior work on quality of life (QOL) in EB has identified significant emotional burden for caregivers of patients with severe EBS, second only to caregivers of patients with recessive dystrophic epidermolysis bullosa (RDEB) [12]. When compared to other skin conditions, EBS patients also report similar levels of health-related impact on QOL as patients with moderate to severe psoriasis and atopic dermatitis [13, 14]. Moreover, even localized EBS, the mildest subtype of EBS, has been shown to impact quality of life due to severe pain during and impaired mobility during blister flares, and remains likely both undertreated and underdiagnosed [1, 14].

Unfortunately, prior research on disease burden in EBS has been significantly limited by small sample sizes. Studies with larger cohorts tend to focus on RDEB [15], even when EBS patients may be eligible. Thus, despite being the most common variant of EB, there is a paucity of literature on the clinical features and patient-reported outcomes associated with EBS. In this cross-sectional survey study, using the largest international cohort of EBS participants to date, we evaluated detailed disease manifestations and health-related impact on QOL in EBS and investigated age-specific differences in clinical outcomes among EBS patients.

## Results

Two hundred and twenty participants completed a survey distributed by the EBCare Registry, an international online EB registry and database, and self-reported a diagnosis of EBS of any subtype. Four respondents were excluded due to duplicate entries or missing data in all fields. Two respondents who self-reported a diagnosis of EBS due to plakophilin deficiency (EBS-PD) were excluded as this was re-classified as a non-EBS skin fragility disorder in the 2014 and 2020 EB consensus reclassification schemes [1, 16].

The final sample consisted of 214 respondents with a self-reported diagnosis of EBS (Table 1). Mean respondent age was 32.8 years (standard deviation [SD] = 19.2). Twenty-nine percent (n = 63) reported diagnostic confirmation of their disease through skin biopsy or genetic testing. Sixty-four percent (n = 137) had a first-degree relative diagnosed with EB, and 36% (n = 77) had a first-degree relative who underwent testing for their EB. Most (93%, n = 200) were from North America or Europe, and the majority of participants came from four countries: the United States (66%, n = 141), United Kingdom (13%, n = 28), Ireland (7%, n = 15), and Canada (5%, n = 10).

**Table 1:** Cohort characteristics of 214 respondents with epidermolysis bullosa simplex (EBS)

Characteristic	All, No. (%) (n = 214)
Mean age (SD)	32.8 (19.2)
< 18 years	59 (27.6)
≥ 18 years	155 (72.4)
Sex	
Male	76 (35.5)
Female	138 (64.5)
Location	
North America	151 (70.6)
Europe	49 (22.9)
Asia/Australia	8 (3.7)
South America	4 (1.9)
Africa	2 (0.9)
Race/Ethnicity	
White (non-Hispanic)	178 (83.2)
Asian	8 (3.7)
Black	5 (2.3)
Hispanic	4 (1.9)
Middle Eastern	2 (0.9)
Multiracial	11 (5.1)
Other or not answered	6 (2.8)
EBS subtype	
Localized	94 (43.9)
Intermediate	9 (4.2)
Severe	26 (12.1)
Other^a^	4 (1.9)
Unsure which EBS subtype	81 (37.9)
Self-reported disease severity	
Mild	82 (38.3)
Moderate	100 (46.7)
Severe	21 (9.8)
Unsure or not answered	11 (5.1)
Age of EBS diagnosis	
Prenatal	8 (3.7)
Birth to 11 months	128 (59.8)
1 to 9 years	45 (21.0)
≥ 10 years	24 (11.2)
Unsure or not answered	9 (4.2)
Method of diagnosis^b^	
Genetic testing	25 (11.7)
Skin biopsy	48 (22.4)
Clinical diagnosis	121 (56.5)
Prenatal testing	3 (1.4)
Family history	11 (5.1)
Other, unsure, or not answered	6 (2.8)
Diagnostic confirmation with genetic testing or skin biopsy^b^	63 (29.4)
Family History of EB	
First-degree relative diagnosed with EB	137 (64.0)
First-degree relative tested for EB	77 (36.0)

*SD* standard deviation

^a^“Other” subtypes include plectin-related intermediate EBS (previously EBS-Ogna), EBS with mottled pigmentation, and EBS with muscular dystrophy

^b^Respondents were able to report multiple methods of diagnosis

### Clinical manifestations of EBS

The most common clinical characteristics reported by participants with EBS were blisters (93%, n = 199), pain (74%, n = 158), itch (55%, n = 118), difficulty walking (44%, n = 94), constipation (43%, n = 91), nail problems (39%, n = 84), and infections (35%, n = 74) (Table 2). The vast majority of respondents (89%, n = 191) reported recurrent wounds, over half (59%, n = 126) had chronic wounds, and 29% (n = 62) had large (> 7.5 cm) wounds. Respondents also noted extensive extracutaneous disease burden, including difficulty swallowing (15%, n = 32), anemia (13%, n = 27), and failure to thrive (6%, n = 12). Mean worst pain over the past 12 months was 6.8 (from 0 to 10 using the Wong-Baker FACES pain scale [17]; SD = 2.8). Respondents reported routine use of non-opiate analgesics (50%, n = 107) and opiates (12%, n = 26), use of non-opiate analgesics (29%, n = 61) and opiates (6%, n = 12) during dressing changes, topical antibiotics (43%, n = 92), and antihistamines (21%, n = 45). These findings were consistent in subgroup analyses restricted to respondents with diagnostic confirmation of their disease (e.g., via genetic testing or skin biopsy) (n = 63 of 214) (Additional file 1: Table S1).

**Table 2:** Disease burden among respondents with epidermolysis bullosa simplex (EBS), by age

	All, No. (%) (n = 214)	Age 0–9, No. (%) (n = 33)	Age 10–17, No. (%) (n = 26)	Age 18 + , No. (%) (n = 155)	*P* Value
*Clinical characteristics*					
EBS subtype					0.03*
Localized	94 (43.9)	9 (27.3)	7 (26.9)	78 (50.3)	
Intermediate	9 (4.2)	2 (6.1)	1 (3.8)	6 (3.9)	
Severe	26 (12.1)	7 (21.2)	6 (23.1)	13 (8.4)	
Other or unsure which EBS subtype^a^	85 (39.7)	15 (45.5)	12 (46.2)	58 (37.4)	
Self-reported disease severity					0.001*
Mild	82 (38.3)	14 (42.4)	11 (42.3)	57 (36.8)	
Moderate	100 (46.7)	8 (24.2)	10 (38.5)	82 (52.9)	
Severe	21 (9.8)	8 (24.2)	5 (19.2)	8 (5.2)	
Not answered	11 (5.1)	3 (9.1)	0 (0.0)	8 (5.2)	
Diagnostic confirmation with genetic testing or skin biopsy	63 (29.4)	16 (48.5)	9 (34.6)	35 (22.6)	< 0.001*
Mean worst pain in past 12 months, ± SD	6.8 ± 2.8	6.5 ± 2.8	6.9 ± 3.3	6.8 ± 2.7	0.64
Mean QOLEB, ± SD^b^	14.7 ± 7.5	15.3 ± 10.3	16.6 ± 7.5	14.3 ± 7.2	0.75
Body mass index^c^					< 0.001*
Underweight	12 (5.6)	1 (3.0)	4 (15.4)	7 (4.5)	
Healthy weight	63 (29.4)	9 (27.3)	11 (42.3)	43 (27.7)	
Overweight/obese	81 (37.9)	2 (6.1)	0 (0.0)	79 (51.0)	
Not answered	58 (27.1)	21 (63.6)	11 (42.3)	26 (16.8)	
Clinical manifestations					
Blisters	199 (93.0)	29 (87.9)	26 (100.0)	144 (92.9)	0.21
Pain	158 (73.8)	22 (66.7)	23 (88.5)	113 (72.9)	0.15
Itch	118 (55.1)	17 (51.5)	19 (73.1)	82 (52.9)	0.15
Difficulty walking	94 (43.9)	8 (24.2)	12 (46.2)	74 (47.7)	0.04*
Constipation	91 (42.5)	15 (45.5)	12 (46.2)	64 (41.3)	0.84
Nail problems	84 (39.3)	15 (45.5)	14 (53.8)	55 (35.5)	0.15
Infections	74 (34.6)	11 (33.3)	13 (50.0)	50 (32.3)	0.24
Dental caries	54 (25.2)	5 (15.2)	11 (42.3)	38 (24.5)	0.07
Ophthalmic problems	34 (15.9)	1 (3.0)	3 (11.5)	30 (19.4)	0.04*
Milia	33 (15.4)	7 (21.2)	5 (19.2)	21 (13.5)	0.40
Difficulty swallowing	32 (15.0)	7 (21.2)	4 (15.4)	21 (13.5)	0.45
Anemia	27 (12.6)	3 (9.1)	3 (11.5)	21 (13.5)	0.94
Difficulty sleeping	25 (11.7)	2 (6.1)	3 (11.5)	20 (12.9)	0.61
Genitourinary problems	17 (7.9)	1 (3.0)	3 (11.5)	13 (8.4)	0.46
Hair problems	17 (7.9)	3 (9.1)	1 (3.8)	13 (8.4)	0.84
Failure to thrive	12 (5.6)	3 (9.1)	4 (15.4)	5 (3.2)	0.02*
Gastrostomy tube	9 (4.2)	5 (15.2)	3 (11.5)	1 (0.6)	< 0.001*
*Wound characteristics*					
Presence of small wounds (< 2.5 cm)^d^	165 (77.1)	28 (84.8)	18 (69.2)	119 (76.8)	0.38
Presence of medium wounds (2.5–7.5 cm)^d^	107 (50.0)	18 (54.5)	13 (50.0)	76 (49.0)	0.86
Presence of large wounds (> 7.5 cm)^d^	62 (29.0)	8 (24.2)	11 (42.3)	43 (27.7)	0.28
Presence of chronic wounds^e^	126 (58.9)	23 (69.7)	18 (69.2)	85 (54.8)	0.15
Anatomic location of chronic wounds^e^					
Head and neck	28 (13.1)	7 (21.2)	2 (7.7)	19 (12.3)	0.30
Upper extremities	37 (17.3)	9 (27.3)	5 (19.2)	23 (14.8)	0.21
Trunk and lower back	8 (3.7)	3 (9.1)	2 (7.7)	3 (1.9)	0.05
Buttocks and genitals	12 (5.6)	5 (15.2)	2 (7.7)	5 (3.2)	0.02*
Lower extremities	116 (54.2)	22 (66.7)	17 (65.4)	17 (11.0)	0.11
Presence of recurrent wounds^f^	191 (89.3)	31 (93.9)	22 (84.6)	138 (89.0)	0.49
Anatomic location of recurrent wounds^f^					
Head and neck	47 (22.0)	12 (36.4)	8 (30.8)	27 (17.4)	0.03*
Upper extremities	88 (41.1)	16 (48.5)	10 (38.5)	62 (40.0)	0.68
Trunk and lower back	19 (8.9)	5 (15.2)	2 (7.7)	12 (7.7)	0.36
Buttocks and genitals	31 (14.5)	8 (24.2)	3 (11.5)	20 (12.9)	0.24
Lower extremities	183 (85.5)	30 (90.9)	22 (84.6)	131 (84.5)	0.71
Number of dressing changes per week					0.16
0 to 3 changes/week	56 (26.2)	11 (33.3)	5 (19.2)	40 (25.8)	
4 to 9 changes/week	80 (37.4)	14 (42.4)	7 (26.9)	59 (38.1)	
10 + changes/week	11 (5.1)	1 (3.0)	4 (15.4)	6 (3.9)	
Not answered	67 (31.3)	7 (21.2)	10 (38.5)	50 (32.3)	
*Medication Use*					
Non-opiate analgesics (routine use)^g^	107 (50.0)	19 (57.6)	14 (53.8)	74 (47.7)	0.55
Topical antibiotics^h^	92 (43.0)	22 (66.7)	18 (69.2)	52 (33.5)	< 0.001*
Non-opiate analgesics (dressing changes)^g^	61 (28.5)	15 (45.5)	8 (30.8)	38 (24.5)	0.05
Antihistamines^i^	45 (21.0)	12 (36.4)	5 (19.2)	28 (18.1)	0.08
Laxatives^j^	34 (15.9)	5 (15.2)	6 (23.1)	23 (14.8)	0.57
Opiates (routine use)^k^	26 (12.1)	7 (21.2)	3 (11.5)	16 (10.3)	0.24
Psychiatric medications^l^	24 (11.2)	0 (0.0)	4 (15.4)	20 (12.9)	0.05*
Topicals steroids^m^	16 (7.5)	4 (12.1)	0 (0.0)	12 (7.7)	0.23
Opiates (dressing changes)^k^	12 (5.6)	3 (9.1)	0 (0.0)	9 (5.8)	0.39
Systemic steroids^n^	6 (2.8)	0 (0.0)	1 (3.8)	5 (3.2)	0.66

Statistical significance was assessed using Kruskal–Wallis tests and Fisher’s exact tests. Respondents who did not answer have been excluded from the comparison. *Significance at the 0.05 level

*SD* standard deviation

^a^“Other” subtypes include plectin-related intermediate EBS (previously EBS-Ogna), EBS with mottled pigmentation, and EBS with muscular dystrophy

^b^Only respondents who completed all 17 items in the QOLEB survey were included. QOLEB scores are stratified as follows: very mild (0–4), mild (5–9), moderate (10–19), severe (20–34), and very severe (35–51)

^c^Body mass index was calculated and categorized based on age-appropriate guidelines for respondents with available height and weight data

^d^Respondents were able to select more than one wound size category

^e^Chronic wounds were defined as “areas that have not healed for weeks/months”

^f^Recurrent wounds were defined as “areas that are difficult to heal”

^g^Non-opiate analgesics: acetaminophen, ibuprofen, naproxen, ketorolac, celecoxib, gabapentin, pregabalin, oxcarbazepine

^h^Topical antibiotics: mupirocin, bacitracin, polymixin, neosporin, gentamicin, retapamulin, chlorhexidine

^i^Antihistamines: diphenhydramine, cetirizine, cyproheptadine, hydroxyzine, loratidine

^j^Laxatives: lactulose, fiber, senna, bisacodyl, polyethylene glycol, magnesium hydroxide, castor oil, docusate

^k^Opiates: codeine, hydrocodone, oxycodone, hydromorphone, fentanyl, methadone, morphine

^l^Psychiatric medications: amitriptyline, amphetamine, aripiprazole, atomoxetine, bupropion, buspirone, carbamazepine, clorazepate, chlordiazepoxide, chlorpromazine, citalopram, dexmethylphenidate, diazepam, doxepin, duloxetine, escitalopram, fluphenazine, fluvoxamine, guanfacine, haloperidol, imipramine, lamotrigine, lithium, lorazepam, lurasidone, methylphenidate, midazolam, mirtazapine, nefazodone, oxcarbazepine, paroxetine, quetiapine, risperidone, sertraline, thioridazine, valproate, venlafaxine

^m^Topical steroids: hydrocortisone, triamcinolone

^n^Systemic steroids: prednisone

Disease manifestations varied between pediatric and adult respondents. Children and adolescents were more likely to be diagnosed with the subtype severe EBS (21% vs. 23% vs. 8% for respondents aged 0–9 vs. 10–17 vs. 18 + , p = 0.03), and to report increased rates of gastrostomy tubes (15% vs. 12% vs. 1%, p < 0.001), failure to thrive (9% vs. 15% vs. 3%, p = 0.02) and use of topical antibiotics (67% vs. 69% vs. 34%, p < 0.001). Routine use of pain medications including opiates was highest during childhood and decreased with age, though these observed relationships were not statistically significant (non-opiate analgesics: 58% vs. 54% vs. 48%, p = 0.55; opiates: 21% vs. 12% vs. 10%, p = 0.24). But, almost half of adolescent and adult respondents reported difficulty walking compared to 24% of pre-pubescent respondents (p = 0.04), and adult respondents were more likely to be overweight or obese (6% vs. 0% vs. 51%, p < 0.001) and to use psychiatric medications (0% vs. 15% vs. 13%, p = 0.05).

We performed sensitivity analyses investigating differences by geographic location to account for potential regional differences. We compared respondent and disease characteristics across the four most represented countries in this cohort (United States, Canada, United Kingdom, and Ireland) as the majority of participants originated from these locations. No significant geographic differences in respondent age or EBS subtype were identified. However, respondents from the United States were more likely to report medication use, including routine use of non-opiate analgesics (60% vs. 40% vs. 39% vs. 13% for the United States vs. Canada vs. United Kingdom vs. Ireland, p = 0.001), and use of topical antibiotics (55% vs. 20% vs. 14% vs. 0%, p < 0.001) and antihistamines (26% vs. 10% vs. 7% vs. 7%, p = 0.047).

### Clinical manifestations by EBS subtype in adults

We observed significant differences across EBS subtypes among adult respondents, including increased incidence of itch (36% vs. 74% in adults with localized EBS vs. intermediate or severe EBS subtypes, p = 0.004), nail problems (23% vs. 63%, p = 0.002), and dental caries (12% vs. 53%, p < 0.001) among respondents with more severe EBS subtypes (Table 3). Respondents with localized EBS were more likely to be overweight or obese (55% vs. 37%, p = 0.03) and, surprisingly, reported worse mean pain scores (from 0 to 10: 7.0 vs. 5.3, p = 0.02).

**Table 3:** Disease burden among respondents with epidermolysis bullosa simplex (EBS) aged 18 + , by EBS subtype

	Localized subtype, No. (%) (n = 78)	Intermediate/Severe subtype, No. (%) (n = 19)	*P* Value
*Clinical characteristics*			
Self-reported disease severity			0.78
Mild	29 (37.2)	7 (36.8)	
Moderate	40 (51.3)	9 (47.4)	
Severe	5 (6.4)	2 (10.5)	
Not answered	4 (5.1)	1 (5.3)	
Diagnostic confirmation with genetic testing or skin biopsy	16 (20.5)	11 (57.9)	0.003*
Mean worst pain in past 12 months, ± SD	7.0 ± 2.6	5.3 ± 2.9	0.02*
Mean QOLEB, ± SD^a^	13.4 ± 6.8	15.8 ± 6.2	0.29
Body mass index^b^			0.03*
Underweight	4 (5.1)	1 (5.3)	
Healthy weight	17 (21.8)	11 (57.9)	
Overweight/obese	43 (55.1)	7 (36.8)	
Not answered	14 (17.9)	0 (0.0)	
Clinical manifestations			
Blisters	70 (89.7)	17 (89.5)	1.00
Pain	52 (66.7)	16 (84.2)	0.17
Itch	28 (35.9)	14 (73.7)	0.004*
Difficulty walking	37 (47.4)	8 (42.1)	0.80
Constipation	29 (37.2)	10 (52.6)	0.30
Nail problems	18 (23.1)	12 (63.2)	0.002*
Infections	19 (24.4)	8 (42.1)	0.16
Dental caries	9 (11.5)	10 (52.6)	< 0.001*
Ophthalmic problems	10 (12.8)	4 (21.1)	0.47
Milia	6 (7.7)	8 (42.1)	0.001*
Difficulty swallowing	6 (7.7)	4 (21.1)	0.10
Anemia	10 (12.8)	3 (15.8)	0.72
Difficulty sleeping	7 (9.0)	2 (10.5)	1.00
Genitourinary problems	5 (6.4)	3 (15.8)	0.19
Hair problems	5 (6.4)	2 (10.5)	0.62
Failure to thrive	2 (2.6)	0 (0.0)	1.00
Gastrostomy tube	1 (1.3)	0 (0.0)	1.00
*Wound characteristics*			
Presence of small wounds (< 2.5 cm)^c^	61 (78.2)	16 (84.2)	0.76
Presence of medium wounds (2.5–7.5 cm)^c^	34 (43.6)	11 (57.9)	0.31
Presence of large wounds (> 7.5 cm)^c^	19 (24.4)	6 (31.6)	0.56
Presence of chronic wounds^d^	38 (48.7)	12 (63.2)	0.31
Anatomic location of chronic wounds^d^			
Head and neck	6 (7.7)	8 (42.1)	0.001*
Upper extremities	7 (9.0)	6 (31.6)	0.02*
Trunk and lower back	1 (1.3)	2 (10.5)	0.10
Buttocks and genitals	1 (1.3)	2 (10.5)	0.10
Lower extremities	36 (46.2)	10 (52.6)	0.62
Presence of recurrent wounds^e^	70 (89.7)	17 (89.5)	1.00
Anatomic location of recurrent wounds^e^			
Head and neck	7 (9.0)	7 (36.8)	0.006*
Upper extremities	33 (42.3)	7 (36.8)	0.80
Trunk and lower back	2 (2.6)	5 (26.3)	0.003*
Buttocks and genitals	5 (6.4)	6 (31.6)	0.007*
Lower extremities	68 (87.2)	15 (78.9)	0.47
Number of dressing changes per week			1.00
0 to 3 changes/week	21 (26.9)	5 (26.3)	
4 to 9 changes/week	29 (37.2)	6 (31.6)	
10 + changes/week	2 (2.6)	0 (0.0)	
Not answered	26 (33.3)	8 (42.1)	
*Medication Use*			
Non-opiate analgesics (routine use)^f^	39 (50.0)	10 (52.6)	1.00
Topical antibiotics^g^	24 (30.8)	7 (36.8)	0.60
Non-opiate analgesics (dressing changes)^f^	23 (29.5)	2 (10.5)	0.14
Antihistamines^h^	8 (10.3)	5 (26.3)	0.12
Laxatives^i^	9 (11.5)	4 (21.1)	0.28
Opiates (routine use)^j^	6 (7.7)	1 (5.3)	1.00
Psychiatric medications^k^	9 (11.5)	3 (15.8)	0.70
Topicals steroids^l^	5 (6.4)	3 (15.8)	0.19
Opiates (dressing changes)^j^	7 (9.0)	0 (0.0)	0.34
Systemic steroids^m^	0 (0.0)	0 (0.0)	1.00

Statistical significance assessed using Kruskal–Wallis tests and Fisher’s exact tests. Respondents who did not answer have been excluded from the comparison. *Significance at the 0.05 level

*SD* standard deviation

^a^Only respondents who completed all 17 items in the QOLEB survey were included. QOLEB scores are stratified as follows: very mild (0–4), mild (5–9), moderate (10–19), severe (20–34), and very severe (35–51)

^b^Body mass index was calculated and categorized based on age-appropriate guidelines for respondents with available height and weight data

^c^Respondents were able to select more than one wound size category

^d^Chronic wounds were defined as “areas that have not healed for weeks/months”

^e^Recurrent wounds were defined as “areas that are difficult to heal”

^f^Non-opiate analgesics: acetaminophen, ibuprofen, naproxen, ketorolac, celecoxib, gabapentin, pregabalin, oxcarbazepine

^g^Topical antibiotics: mupirocin, bacitracin, polymixin, neosporin, gentamicin, retapamulin, chlorhexidine

^h^Antihistamines: diphenhydramine, cetirizine, cyproheptadine, hydroxyzine, loratidine

^i^Laxatives: lactulose, fiber, senna, bisacodyl, polyethylene glycol, magnesium hydroxide, castor oil, docusate

^j^Opiates: codeine, hydrocodone, oxycodone, hydromorphone, fentanyl, methadone, morphine

^k^Psychiatric medications: amitriptyline, amphetamine, aripiprazole, atomoxetine, bupropion, buspirone, carbamazepine, clorazepate, chlordiazepoxide, chlorpromazine, citalopram, dexmethylphenidate, diazepam, doxepin, duloxetine, escitalopram, fluphenazine, fluvoxamine, guanfacine, haloperidol, imipramine, lamotrigine, lithium, lorazepam, lurasidone, methylphenidate, midazolam, mirtazapine, nefazodone, oxcarbazepine, paroxetine, quetiapine, risperidone, sertraline, thioridazine, valproate, venlafaxine

^l^Topical steroids: hydrocortisone, triamcinolone

^m^Systemic steroids: prednisone

### Disease-related impact on quality of life in EBS

A subset of respondents (32.7%, n = 70 of 214) completed all items in the validated Quality of Life in Epidermolysis Bullosa (QOLEB) survey [18] and were included in analyses investigating disease-related impact on QOL (Table 4). Among these respondents, mean QOLEB score was 14.7 (SD = 7.5), indicating a “moderate” impact of EBS on overall QOL [19]. EBS negatively affected daily function, with 41% (n = 29 of 70) of respondents reporting “frequent” or “constant” pain. The majority had functional limitations including mobility restrictions due to EBS: 93% (n = 65 of 70) were unable to participate in sports, 79% (n = 55 of 70) reported difficulty moving outside their home, and 70% (n = 49 of 70) reported difficulty moving at home. Most respondents also experienced emotional and psychosocial distress due to their EBS including feelings of frustration (99%, n = 69 of 70), anxiety (70%, n = 49 of 70), embarrassment (66%, n = 46 of 70), discomfort due to teasing or staring (66%, n = 46 of 70), and depression (54%, n = 38 of 70).

**Table 4:** QOLEB measures in respondents with epidermolysis bullosa simplex (EBS), by age

QOLEB measure	All, No (%) (n = 70)	Age 0–17, No. (%) (n = 14)	Age 18 + , No. (%) (n = 56)	P value
Ability to move around at home				0.12
No impact	21 (30.0)	5 (35.7)	16 (28.6)	
A little	39 (55.7)	7 (50.0)	32 (57.1)	
A lot	10 (14.3)	2 (14.3)	8 (14.3)	
Severely impacted	0 (0.0)	0 (0.0)	0 (0.0)	
Ability to bath or shower				0.94
No impact	56 (80.0)	11 (78.6)	45 (80.4)	
Sometimes need assistance	13 (18.6)	3 (21.4)	10 (17.9)	
Need assistance most of the time	0 (0.0)	0 (0.0)	0 (0.0)	
Need assistance every time	1 (1.4)	0 (0.0)	1 (1.8)	
Physical pain				0.59
No pain	4 (5.7)	0 (0.0)	4 (7.1)	
Occasional pain	37 (52.9)	10 (71.4)	27 (48.2)	
Frequent pain	25 (35.7)	3 (21.4)	22 (39.3)	
Constant pain	4 (5.7)	1 (7.1)	3 (5.4)	
Ability to write				0.89
No impact	49 (70.0)	10 (71.4)	39 (69.6)	
Difficult to grip pen	10 (14.3)	2 (14.3)	8 (14.3)	
Easier to type than write	10 (14.3)	2 (14.3)	8 (14.3)	
Cannot write due to EB	1 (1.4)	0 (0.0)	1 (1.8)	
Ability to eat				0.37
No impact	58 (82.9)	10 (71.4)	48 (85.7)	
A little	9 (12.9)	2 (14.3)	7 (12.5)	
A lot	2 (2.9)	2 (14.3)	0 (0.0)	
Rely on gastrostomy tube for nutrition	1 (1.4)	0 (0.0)	1 (1.8)	
Ability to go shopping				0.6
No impact	18 (25.7)	3 (21.4)	15 (26.8)	
A little	40 (57.1)	8 (57.1)	32 (57.1)	
A lot	10 (14.3)	2 (14.3)	8 (14.3)	
Need assistance every time	2 (2.9)	1 (7.1)	1 (1.8)	
Involvement in sports				0.25
No impact	5 (7.1)	1 (7.1)	4 (7.1)	
Need to be cautious in sports	17 (24.3)	1 (7.1)	16 (28.6)	
Need to avoid some sports	48 (68.6)	12 (85.7)	36 (64.3)	
Need to avoid all sports	0 (0.0)	0 (0.0)	0 (0.0)	
Feelings of frustration				0.92
None	1 (1.4)	0 (0.0)	1 (1.8)	
A little	34 (48.6)	7 (50.0)	27 (48.2)	
A lot	31 (44.3)	6 (42.9)	25 (44.6)	
Severe/constant	4 (5.7)	1 (7.1)	3 (5.4)	
Ability to move around outside of home				0.72
No impact	15 (21.4)	4 (28.6)	11 (19.6)	
A little	29 (41.4)	6 (42.9)	23 (41.1)	
A lot	22 (31.4)	1 (7.1)	21 (37.5)	
Severely impacted	4 (5.7)	3 (21.4)	1 (1.8)	
Impact on relationships with family members				0.15
No impact	27 (38.6)	3 (21.4)	24 (42.9)	
A little	33 (47.1)	8 (57.1)	25 (44.6)	
A lot	9 (12.9)	2 (14.3)	7 (12.5)	
Severely impacted	1 (1.4)	1 (7.1)	0 (0.0)	
Feelings of embarrassment				0.34
None	24 (34.3)	7 (50.0)	17 (30.4)	
A little	28 (40.0)	4 (28.6)	24 (42.9)	
A lot	10 (14.3)	1 (7.1)	9 (16.1)	
Severe/constant	8 (11.4)	2 (14.3)	6 (10.7)	
Home modifications (e.g., installing ramps) due to EB				0.69
None	64 (91.4)	12 (85.7)	52 (92.9)	
A few	5 (7.1)	2 (14.3)	3 (5.4)	
A lot	1 (1.4)	0 (0.0)	1 (1.8)	
Extensive	0 (0.0)	0 (0.0)	0 (0.0)	
Impact on relationships with friends				0.37
No impact	32 (45.7)	5 (35.7)	27 (48.2)	
A little	28 (40.0)	6 (42.9)	22 (39.3)	
A lot	7 (10.0)	2 (14.3)	5 (8.9)	
Severely impacted	3 (4.3)	1 (7.1)	2 (3.6)	
Feelings of anxiety or worry				0.84
None	21 (30.0)	5 (35.7)	16 (28.6)	
A little	31 (44.3)	4 (28.6)	27 (48.2)	
A lot	11 (15.7)	3 (21.4)	8 (14.3)	
Severe/constant	7 (10.0)	2 (14.3)	5 (8.9)	
Financial impact of EB				0.2
No impact	15 (21.4)	4 (28.6)	21 (37.5)	
Slightly impacted	32 (45.7)	5 (35.7)	27 (48.2)	
Greatly impacted	12 (17.1)	4 (28.6)	8 (14.3)	
Severely impacted	1 (1.4)	1 (7.1)	0 (0.0)	
Feelings of depression				0.31
None	32 (45.7)	5 (35.7)	27 (48.2)	
A little	25 (35.7)	5 (35.7)	20 (35.7)	
A lot	11 (15.7)	3 (21.4)	8 (14.3)	
Severe/constant	2 (2.9)	1 (7.1)	1 (1.8)	
Feelings of discomfort due to others (e.g., teasing or staring)				0.6
None	24 (34.3)	7 (50.0)	17 (30.4)	
A little	32 (45.7)	3 (21.4)	29 (51.8)	
A lot	13 (18.6)	4 (28.6)	9 (16.1)	
Severe/constant	1 (1.4)	0 (0.0)	1 (1.8)	

Only respondents who self-reported disease severity and who completed all 17 items of the QOLEB survey were included. Statistical significance assessed using Fisher's exact test. *Significance at the 0.05 level

In analyses among respondents who completed all items in the QOLEB survey (n = 70 of 214), QOL did not vary significantly by respondent age (QOLEB score: 14.7 vs. 15.3 vs. 16.6 for respondents aged 0–9 vs. 10–17 vs. 18 + , p = 0.75). However, in subgroup analyses restricted to respondents who both completed the full QOLEB survey and reported diagnostic confirmation via genetic testing or skin biopsy (n = 19 of 214), we observed worse QOL in adolescent and adult respondents (QOLEB score: 6.7 vs. 20.8 vs. 14.3, p = 0.03). Worse QOL also correlated with more severe EBS subtypes, though this association did not reach statistical significance (QOLEB score: 13.4 vs. 15.8 for respondents with localized EBS vs. intermediate or severe EBS subtypes, p = 0.29).

## Discussion

In this study, we report the most common clinical symptoms and manifestations associated with EBS using cross-sectional survey data captured from a global cohort of 214 EBS patients, the largest international sample of EBS patients to date. Our findings run counter to the notion of EBS as simply a mild form of EB, and instead demonstrate that EBS patients experience significant disease burden including pain and itch, functional impairments, and reduced QOL.

Wound burden in EBS can be extensive. In this cohort, 29% had large wounds, which was comparable to the prevalence of large wounds reported among patients with dominant dystrophic EB (DDEB) [20] and approximately half the prevalence observed in patients with RDEB [21]. Seventy-four percent and 55% of respondents reported pain and itch, respectively, suggesting significant wound-associated morbidity. Mean worst pain was reported as 6.8 out of 10 among all respondents, and 12% of respondents routinely used opiates, which was four times greater than the rate of opiate use observed among DDEB patients [20], and comparable to rates of opiate use in patients with mild RDEB [21]. We observed a decline in the use of opiate and non-opiate analgesics with increased age both for routine pain management and for pain associated with dressing changes (though these associations were not statistically significant), which supports prior observations that the severity of blistering and pain in EBS may improve as patients grow older [10, 22].

These clinical manifestations may contribute to functional impairments including difficulties with ambulation and physical activity. In EBS, causes of impaired mobility are multifactorial and may evolve with age. Physical activity during childhood is primarily limited by pain from blisters on the feet. Over time, however, repeated cycles of wounding and healing can result in the development of plantar keratoderma, producing a cycle of increased pain followed by reduced physical activity and increased body weight as patients grow older [11]. Seasonal variation in mobility due to fluctuations in blistering and pain have also been identified, with symptoms often flaring in the summer due to increased heat and humidity [10, 14]. In this study, almost half of adolescents and adults in our cohort had difficulty walking compared to just 24% of children (p = 0.04). These findings demonstrate the age-specific impact of EBS on walking, and illustrate that while blistering frequency and severity may improve with age for some EBS patients [10, 22], mobility impairments can persist and even worsen during adulthood. As observed in our cohort, adults with localized EBS may also experience worse pain compared to those with intermediate or severe EBS subtypes. This may be due to the distribution of blisters and erosions primarily on the palms and soles in localized EBS, which can result in severe pain and significant impairment of QOL and daily activities including regular attendance of school or full-time work [10, 14].

Patients with EBS can also develop extracutaneous disease manifestations including difficulties with eating and drinking due to blistering and ulceration of the oral mucosa [23]. Gastrointestinal involvement is primarily considered a characteristic of other forms of inherited EB, including dystrophic EB [24]. In this cohort, however, 15% of respondents under nine years of age and 12% of respondents aged ten to seventeen required gastrostomy tubes, demonstrating that a small but significant subset of participants—in particular, younger EBS patients—may experience dysphagia and require extensive nutritional support [23, 25–27]. Critically, the natural history of body mass and weight in EBS is distinct from that observed in other EB subtypes. Individuals with EBS are more likely to be underweight during childhood (a feature common to other EB subtypes) but are, uniquely, at risk of becoming overweight or obese during adolescence and adulthood [23]. Unfortunately, there are currently no clear guidelines on the prevention and management of obesity within this patient population, and further work is needed to clarify associated risks, comorbidities, and consequences of obesity in EBS.

Within our cohort, the overall health-related impact of EBS on QOL was “moderate” [19], comparable to patients with mild RDEB in a similar study [21]. The emotional and QOL burden present in this cohort—including the large proportion of respondents identifying feelings of frustration and embarrassment, and impaired participation in physical activities—are consistent with prior work assessing wellbeing in all EB subtypes [28]. However, much remains unknown about psychosocial health in EBS and how this may differ from patients with other forms of inherited EB [28, 29].

Notably, the emotional and psychosocial challenges of EBS may change by age. We did not observe any age-specific differences in QOL within this cohort, although this may have been limited by the small sample size of respondents who completed the full QOLEB survey. Prior work have identified that children with EBS can experience difficulties developing and maintaining friendships, and encounter restrictions on routine physical activities including play due to the risk of blistering on the hands from mechanical and frictional trauma [13, 30, 31]. One previous study also found that pediatric patients may encounter skepticism and a lack of empathy from peers who do not understand the fluctuating nature of EBS [32]. These difficulties may persist into adulthood, particularly among individuals unable to fully participate in work or social activities, and can contribute to worsening physical manifestations of disease including obesity due to social isolation and depression [11, 14, 29]. In the present study, 15% and 13% of adolescent and adult respondents used psychiatric medications, respectively. Unfortunately, little is known about specific psychiatric comorbidities associated with EBS and there have been no investigations of concrete interventions to provide emotional and psychosocial support for EBS patients and their families, particularly as patients transition into adulthood [28]. Our findings affirm the ongoing need for research on interventions to guide management of EBS-related psychosocial problems [28], including strategies to facilitate participation in physical and social activities for both children and adults with EBS.

Patient-reported outcomes including those captured in the EBCare registry are increasingly recognized as critical to clinical trials, as they incorporate the patient’s own perspective and lived experience of their disease and treatment course [33]. Clinical trials investigating novel therapeutics should focus on major sources of disease burden in EBS including pain, itch, and mobility restrictions given the significant proportion of patients affected by these symptoms, and trials which include EBS patients should consider age stratification given the natural history of EBS and variation in disease outcomes by age. Furthermore, this study quantifies pain and QOL in EBS, and the mean and variance of these important endpoints will help improve the design and powering of future clinical trials in EBS.

This study has several limitations including recall bias and missing data due to the patient-reported survey study design. Because this survey was anonymized, we were unable to verify the survey results, such as diagnosis of EBS, clinical symptoms and outcomes, wound characteristics, and method of diagnosis. We were also unable to assess longitudinal changes in clinical manifestations and patient-reported outcomes due to the cross-sectional design of this survey. Subjective clinical manifestations such as pain severity and disease-related emotional burden can also be influenced by non-EBS factors including respondent beliefs, attitudes, age, and cultural background, which may limit the generalizability of these results. This survey also does not address caregiver burden as investigated in other related studies [34], which limits our ability to capture the impact of EBS on the psychosocial wellbeing of the entire family unit. Moreover, we could not estimate a survey response rate as participants were recruited online or at Dystrophic Epidermolysis Bullosa Research Association (DEBRA) of America meetings. EBS can be difficult to distinguish from other EB subtypes and only a subset of respondents received diagnostic confirmation of their EBS diagnosis, likely due to the infrequency of genetic testing during the survey collection timeframe compared to the present day [35]. To account for the possibility of misdiagnosis, we performed subgroup analyses on respondents who self-reported diagnostic confirmation via gene testing or skin biopsy. Those EBS subjects with genetic or biopsy confirmation (n = 63) had the same common clinical symptoms as the full EBS cohort (n = 214) suggesting that our conclusions are valid. Lastly, we acknowledge that the data are taken from a survey conducted more than five years ago; however, this remains the largest dataset of clinical manifestations and health-related impacts on QOL among EBS patients to date. Future studies should be conducted prospectively to incorporate our more recent understanding of EBS, including investigations of treatments to reduce pain and itch, as well as dedicated psychosocial interventions to support psychosocial wellbeing and QOL in EBS.

## Capsule summary

- In this global, cross-sectional survey of 214 survey respondents with EBS, extensive disease burden including blisters, pain, itch, difficulty walking were observed.
- Disease burden varied by patient age. Pediatric respondents were more likely to require gastrostomy tubes for nutritional support and to report failure to thrive, while adolescent and adult respondents were more likely to have difficulty walking, which may contribute to the increased rate of obesity seen among adults.
- These disease manifestations may affect both the emotional wellbeing and functional ability of EBS patients, with most respondents reporting feelings of frustration, embarrassment, anxiety, and depression, as well as restricted mobility due to their EBS.
- Further research investigating medical treatments and age-appropriate psychosocial interventions to improve clinical outcomes and QOL in EBS are needed.

## Conclusion

In this study, we characterized patient-reported outcomes and QOL in 214 EBS survey respondents. Patients with EBS experience extensive disease burden including significant large wounds comparable to other forms of inherited EB, pain, itch, and functional impairments. Disease manifestations in EBS may evolve with age. Further research and the development of dedicated interventions to improve clinical outcomes and QOL in EBS are needed.

## Methods

### Study aim, design, and outcomes

The aim of this study was to investigate the most common patient-reported clinical manifestations and the health-related impact of QOL in EBS, and to examine age-specific differences in EBS disease characteristics and burden. Our data come from a global cross-sectional survey of patients with EB, or their caregivers, who were invited online and at DEBRA of America meetings to complete this survey. The survey was conducted between February 2012 and October 2016 by the EBCare Registry, an online international registry and database created by DEBRA of America, DEBRA International, and Lotus Tissue Repair, Inc. (now Phoenix Tissue Repair, Inc., Boston, MA), and managed by Invitae (San Francisco, CA). Respondents self-reporting a diagnosis of EBS were included. This study was exempt from review by the Stanford University Institutional Review Board as the data do not contain any identifying information.

The survey was comprised of two sections: the Medical Profile Survey, and the QOLEB survey. The Medical Profile Survey captured respondent-reported data on demographics, EB diagnosis, clinical features, wound characteristics, and medication use. Diagnostic confirmation was defined as respondent self-report of prior genetic testing or skin biopsy. Self-reported disease severity was determined by degree of symptoms and the impact of skin lesions on activities of daily living (ADLs): “mild” (respondent has noticeable symptoms but ADLs remain unaffected), “moderate” (respondent uses treatment for symptoms and ADLs are frequently affected by skin lesions), and “severe” (respondent consistently requires treatment for symptoms and they are unable to perform ADLs). For respondents who reported height and weight, we calculated body mass index based on age-specific guidelines and growth charts from the United States Centers for Disease Control and Prevention [36]. Respondents reported wound characteristics including the anatomic locations of wounds, wound size, and presence of recurrent or chronic wounds. Chronic wounds were defined as “areas that have not healed for weeks/months” and recurrent wounds were defined as “areas that are difficult to heal” [21, 37]. Respondents reported the worst pain experienced in the past year using the Wong-Baker FACES pain scale [17].

QOLEB is a validated survey which assesses the specific impact of EB across multiple domains of QOL, including ability to perform daily tasks, interpersonal relationships, and emotional burden [18]. The QOLEB survey consists of 17 questions scored on a four-point scale from 0 (no impact on QOL) to 3 (severe impact on QOL). The final QOLEB score was calculated by summing all of a respondents’ answers and interpreted in clinically-relevant strata consistent with prior work on quality of life in EB patients: very mild (0–4 points), mild (5–9 points), moderate (10–19 points), severe (20–34 points), and very severe (35–51 points) [19]. Only respondents who completed all 17 items in the QOLEB survey were included in analyses investigating QOL (n = 70 of 214).

### Statistical analysis

Descriptive statistics were calculated as means, standard deviations, and ranges for continuous variables, and percentages for categorical variables. To assess differences in clinical features across age groups, we performed Kruskal–Wallis tests for continuous variables and Fisher’s exact tests for categorical variables. To account for possible misdiagnosis of EBS in patients without confirmatory diagnostic testing, we performed subgroup analyses to investigate clinical manifestations among respondents with diagnostic confirmation of their EBS diagnosis via genetic testing and skin biopsy (n = 63 of 214). These results were then compared to the group as a whole. We additionally investigated differences in clinical manifestations by geographic location, and by EBS subtype among respondents aged 18 or older. All statistical tests were two-sided, and *p* < 0.05 was significant. Analyses were performed in Stata/SE 16.1 (College Station, TX) and SAS version 9.4 (SAS Institute Inc., Cary, NC).

## Supplementary Information

**Additional file 1. Table S1**: Disease burden among respondents with epidermolysis bullosa simplex reporting diagnostic confirmation, by age.

## Data Availability

The deidentified data that support the findings of this study are available from the Epidermolysis Bullosa Research Partnership.
