# Supplementary material for: A global, cross-sectional survey of patient-reported outcomes, disease burden, and quality of life in epidermolysis bullosa simplex
Source: Orphanet J Rare Dis. 2022 Jul 15;17:270. doi: 10.1186/s13023-022-02433-3 (PMC9287948; doi:10.1186/s13023-022-02433-3)
Supplement: Supplementary file 1 — Additional file 1. Table S1: Disease burden among respondents with epidermolysis bullosa simplex reporting diagnostic confirmation, by age. [file 13023_2022_2433_MOESM1_ESM.docx]

**Supplemental Table 1: Disease burden among respondents with epidermolysis bullosa simplex (EBS) reporting diagnostic confirmation, by age^[a]^**

|  | **All,**  **No. (%) (n=63)** | **Age 0-9, No. (%) (n=16)** | **Age 10-17,**  **No. (%) (n=10)** | **Age 18+, No. (%) (n=37)** | ***P* Value** |
| --- | --- | --- | --- | --- | --- |
| ***Clinical Characteristics*** |  |  |  |  |  |
| EBS subtype |  |  |  |  | 0.71 |
| Localized | 23 (36.5) | 4 (25.0) | 3 (30.0) | 16 (43.2) |  |
| Intermediate | 2 (3.2) | 1 (6.3) | 0 (0.0) | 1 (2.7) |  |
| Severe | 18 (28.6) | 4 (25.0) | 4 (40.0) | 10 (27.0) |  |
| Other or unsure which EBS subtype^[b]^ | 20 (31.7) | 7 (43.8) | 3 (30.0) | 10 (27.0) |  |
| Self-reported disease severity |  |  |  |  | 0.08 |
| Mild | 23 (36.5) | 7 (43.8) | 4 (40.0) | 12 (32.4) |  |
| Moderate | 22 (34.9) | 2 (12.5) | 2 (20.0) | 18 (48.6) |  |
| Severe | 13 (20.6) | 4 (25.0) | 4 (40.0) | 5 (13.5) |  |
| Not answered | 5 (7.9) | 3 (18.8) | 0 (0.0) | 2 (5.4) |  |
| Mean worst pain in past 12 months, ± SD | 6.6 ± 3.1 | 6.5 ± 2.9 | 6.3 ± 4.1 | 6.7 ± 3.0 | 0.94 |
| Mean QOLEB, ± SD^[c]^ | 14.4 ± 7.4 | 6.7 ± 3.2 | 20.8 ± 7.5 | 14.3 ± 6.3 | 0.03* |
| Body mass index^[d]^ |  |  |  |  | 0.007* |
| Underweight | 5 (7.9) | 1 (6.3) | 3 (30.0) | 1 (2.7) |  |
| Healthy weight | 22 (34.9) | 4 (25.0) | 3 (30.0) | 15 (40.5) |  |
| Overweight/obese | 17 (27.0) | 1 (6.3) | 0 (0.0) | 16 (43.2) |  |
| Not answered | 19 (30.2) | 10 (62.5) | 4 (40.0) | 5 (13.5) |  |
| Clinical manifestations |  |  |  |  |  |
| Blisters | 60 (95.2) | 14 (87.5) | 10 (100.0) | 36 (97.3) | 0.22 |
| Pain | 49 (77.8) | 12 (75.0) | 7 (70.0) | 30 (81.1) | 0.70 |
| Itch | 38 (60.3) | 6 (37.5) | 8 (80.0) | 24 (64.9) | 0.07 |
| Difficulty walking | 24 (38.1) | 4 (25.0) | 5 (50.0) | 15 (40.5) | 0.39 |
| Constipation | 25 (39.7) | 3 (18.8) | 7 (70.0) | 15 (40.5) | 0.04* |
| Nail problems | 32 (50.8) | 8 (50.0) | 7 (70.0) | 17 (45.9) | 0.44 |
| Infections | 26 (41.3) | 4 (25.0) | 6 (60.0) | 16 (43.2) | 0.20 |
| Dental caries | 19 (30.2) | 2 (12.5) | 4 (40.0) | 13 (35.1) | 0.19 |
| Ophthalmic problems | 11 (17.5) | 0 (0.0) | 1 (10.0) | 10 (27.0) | 0.04* |
| Milia | 15 (23.8) | 4 (25.0) | 3 (30.0) | 8 (21.6) | 0.85 |
| Difficulty swallowing | 12 (19.0) | 3 (18.8) | 2 (20.0) | 7 (18.9) | 1.00 |
| Anemia | 9 (14.3) | 1 (6.3) | 3 (30.0) | 5 (13.5) | 0.27 |
| Difficulty sleeping | 10 (15.9) | 0 (0.0) | 3 (30.0) | 7 (18.9) | 0.05* |
| Genitourinary problems | 7 (11.1) | 1 (6.3) | 2 (20.0) | 4 (10.8) | 0.64 |
| Hair problems | 7 (11.1) | 2 (12.5) | 1 (10.0) | 4 (10.8) | 1.00 |
| Failure to thrive | 5 (7.9) | 1 (6.3) | 3 (30.0) | 1 (2.7) | 0.03* |
| Gastrostomy tube | 5 (7.9) | 3 (18.8) | 2 (20.0) | 0 (0.0) | 0.01* |
| ***Wound Characteristics*** |  |  |  |  |  |
| Presence of small wounds (<2.5 cm)^[e]^ | 47 (74.6) | 12 (75.0) | 7 (70.0) | 28 (75.7) | 0.92 |
| Presence of medium wounds (2.5-7.5 cm)^[e]^ | 32 (50.8) | 7 (43.8) | 6 (60.0) | 19 (51.4) | 0.73 |
| Presence of large wounds (>7.5 cm)^[e]^ | 21 (33.3) | 5 (31.3) | 6 (60.0) | 10 (27.0) | 0.14 |
| Presence of chronic wounds^[f]^ | 38 (60.3) | 7 (43.8) | 9 (90.0) | 22 (59.5) | 0.94 |
| Anatomic location of chronic wounds^[f]^ |  |  |  |  |  |
| Head and neck | 15 (23.8) | 3 (18.8) | 1 (10.0) | 11 (29.7) | 0.46 |
| Upper extremities | 15 (23.8) | 4 (25.0) | 3 (30.0) | 8 (21.6) | 0.85 |
| Trunk and lower back | 4 (6.3) | 1 (6.3) | 1 (10.0) | 2 (5.4) | 0.79 |
| Buttocks and genitals | 7 (11.1) | 3 (18.8) | 1 (10.0) | 3 (8.1) | 0.55 |
| Lower extremities | 34 (54.0) | 9 (56.3) | 6 (60.0) | 19 (51.4) | 0.88 |
| Presence of recurrent wounds^[g]^ | 57 (90.5) | 14 (87.5) | 9 (90.0) | 34 (91.9) | 0.84 |
| Anatomic location of recurrent wounds^[g]^ |  |  |  |  |  |
| Head and neck | 27 (42.9) | 7 (43.8) | 5 (50.0) | 15 (40.5) | 0.94 |
| Upper extremities | 27 (42.9) | 7 (43.8) | 4 (40.0) | 16 (43.2) | 1.00 |
| Trunk and lower back | 11 (17.5) | 3 (18.8) | 1 (10.0) | 7 (18.9) | 0.91 |
| Buttocks and genitals | 15 (23.8) | 4 (25.0) | 3 (30.0) | 8 (21.6) | 0.85 |
| Lower extremities | 55 (87.3) | 13 (81.3) | 9 (90.0) | 33 (89.2) | 0.77 |
| Number of dressing changes per week |  |  |  |  | 0.07 |
| 0 to 3 changes/week | 19 (30.2) | 5 (31.3) | 4 (40.0) | 10 (27.0) |  |
| 4 to 9 changes/week | 22 (34.9) | 7 (43.8) | 1 (10.0) | 14 (37.8) |  |
| 10+ changes/week | 5 (7.9) | 1 (6.3) | 3 (30.0) | 1 (2.7) |  |
| Not answered | 17 (27.0) | 3 (18.8) | 2 (20.0) | 12 (32.4) |  |
| ***Medication Use*** |  |  |  |  |  |
| Non-opiate analgesics (routine use)^[h]^ | 34 (54.0) | 9 (56.3) | 5 (50.0) | 20 (54.1) | 1.00 |
| Topical antibiotics^[i]^ | 32 (50.8) | 9 (56.3) | 8 (80.0) | 15 (40.5) | 0.07 |
| Non-opiate analgesics (dressing changes)^[h]^ | 19 (30.2) | 7 (43.8) | 3 (30.0) | 9 (24.3) | 0.41 |
| Antihistamines^[j]^ | 18 (28.6) | 5 (31.3) | 3 (30.0) | 10 (27.0) | 0.93 |
| Laxatives^[k]^ | 9 (14.3) | 1 (6.3) | 2 (20.0) | 6 (16.2) | 0.68 |
| Opiates (routine use)^[l]^ | 13 (20.6) | 6 (37.5) | 2 (20.0) | 5 (13.5) | 0.13 |
| Psychiatric medications^[m]^ | 11 (17.5) | 0 (0.0) | 3 (30.0) | 8 (21.6) | 0.06 |
| Topicals steroids^[n]^ | 7 (11.1) | 2 (12.5) | 0 (0.0) | 5 (13.5) | 0.73 |
| Opiates (dressing changes)^[l]^ | 6 (9.5) | 3 (18.8) | 0 (0.0) | 3 (8.1) | 0.34 |
| Systemic steroids^[o]^ | 1 (1.6) | 0 (0.0) | 0 (0.0) | 1 (2.7) | 1.00 |

SD = standard deviation.

[a] Statistical significance assessed using Kruskal-Wallis tests and Fisher’s exact tests. Respondents who did not answer have been excluded from the comparison. * indicates significance at the 0.05 level.

[b] “Other” subtypes include plectin-related intermediate EBS (previously EBS-Ogna), EBS with mottled pigmentation, and EBS with muscular dystrophy.

[c] Only respondents who completed all 17 items in the QOLEB survey were included. QOLEB scores are stratified as follows: very mild (0-4), mild (5-9), moderate (10-19), severe (20-34), and very severe (35-51).

[d] Body mass index was calculated and categorized based on age-appropriate guidelines for respondents with available height and weight data.

[e] Respondents were able to select more than one wound size category.

[f] Chronic wounds were defined as “areas that have not healed for weeks/months.”

[g] Recurrent wounds were defined as “areas that are difficult to heal.”

[h] Non-opiate analgesics: acetaminophen, ibuprofen, naproxen, ketorolac, celecoxib, gabapentin, pregabalin, oxcarbazepine

[i] Topical antibiotics: mupirocin, bacitracin, polymixin, neosporin, gentamicin, retapamulin, chlorhexidine

[j] Antihistamines: diphenhydramine, cetirizine, cyproheptadine, hydroxyzine, loratidine

[k] Laxatives: lactulose, fiber, senna, bisacodyl, polyethylene glycol, magnesium hydroxide, castor oil, docusate

[l] Opiates: codeine, hydrocodone, oxycodone, hydromorphone, fentanyl, methadone, morphine

[m] Psychiatric medications: amitriptyline, amphetamine, aripiprazole, atomoxetine, bupropion, buspirone, carbamazepine, clorazepate, chlordiazepoxide, chlorpromazine, citalopram, dexmethylphenidate, diazepam, doxepin, duloxetine, escitalopram, fluphenazine, fluvoxamine, guanfacine, haloperidol, imipramine, lamotrigine, lithium, lorazepam, lurasidone, methylphenidate, midazolam, mirtazapine, nefazodone, oxcarbazepine, paroxetine, quetiapine, risperidone, sertraline, thioridazine, valproate, venlafaxine

[n] Topical steroids: hydrocortisone, triamcinolone

[o] Systemic steroids: prednisone
